# Coverage and factors associated with completion of continuum of care for maternal health in sub-Saharan Africa: a multicountry analysis

**DOI:** 10.1186/s12884-022-04757-1

**Published:** 2022-05-19

**Authors:** Adugnaw Zeleke Alem, Kegnie Shitu, Tesfa Sewunet Alamneh

**Affiliations:** 1 Department of Epidemiology and Biostatistics, Institute of Public Health, College of Medicine and Health Sciences, University of Gondar, Gondar, Ethiopia; 2 Department of Health Education and Behavioral Science, Institute of Public Health, College of Medicine and Health Sciences, University of Gondar, Gondar, Ethiopia

**Keywords:** Continuum of care, Maternal health care utilization, Sub-Saharan Africa, Multi-country analysis

## Abstract

**Background:**

Many maternal and neonatal deaths are largely preventable by expanding the continuum of care (at least four antenatal visits, skilled birth attendance and postnatal care). Even though ensuring the Continuum of Care (CoC) has advantages over separate services, evidence from the globe suggests that completion of the CoC for maternal health is very low. From our search of the literature, there is limited evidence on the completion of the entire CoC and its associated factors in sub-Saharan Africa (sSA). Therefore, this study aimed to assess coverage and associated factors of completion of the CoC for maternal health in sSA.

**Methods:**

Data for the study were drawn from a recent nationally representative survey of 32 Demographic and Health Surveys (DHS). A total weighted sample of 225,135 women of reproductive-age, who gave birth in the two preceding years were included. Due to the hierarchical nature of DHS data, a multilevel logistic regression model was applied to investigate individual and community-level factors that may influence completion of CoC. Adjusted Odds Ratios (aORs) with 95% Confidence Interval (CI) were reported and variables with 95% CI not including 1 were considered as significant factors of the completion of CoC.

**Results:**

Only, 56,172 (25.0%; 95% CI, 20.5%, 29.4%) of the women in sSA utilized the CoC for maternal health which varied from 11,908 (17.9.0%) in East Africa to 7,418 (51.5% in Southern Africa. Factors associated with higher odds of CoC were women aged 24–34 years (aOR 1.22, 95% CI: 1.17, 1.25), aged ≥ 35 years (aOR 1.40, 95% CI: 1.35, 1.47), attending primary education (aOR 1.44, 95% CI: 1.41, 1.49), secondary education (aOR 1.95, 95% CI: 1.89, 2.03), higher education (aOR 2.15, 95% CI: 2.01, 2.25), having mass media exposure (aOR 1.35, 95% CI: 1.28, 1.39), women from female-headed households (aOR 1.18, 95% CI: 1.15, 1.21) and women from communities with high maternal education (aOR 1.12, 95% CI: 1.09, 1.16). However, perceiving distance from the health facility as a big problem (aOR 0.88, 95% CI: 0.85, 0.91), residing in rural areas (aOR 0.78, 95% CI: 0.75, 0.81), delayed ANC initiation (aOR 0.43, 95% CI: 0.41, 0.47) and unintended pregnancy (aOR 0.87, 95% CI: 0.84, 0.91) were associated with lower odds of CoC.

**Conclusion:**

This study showed a low proportion of women, who utilized the CoC in sSA. Both individual and community-level factors were associated with CoC completion rates among women in sSA. Therefore, policymakers in sSA must consider both individual and community-level factors and undertake multi-sectorial approaches to address barriers of CoC at different levels.

**Supplementary Information:**

The online version contains supplementary material available at 10.1186/s12884-022-04757-1.

## Background

Although the Maternal Mortality Ratio (MMR) declined from 342 per 100,000 live births in 2000 to 211 deaths per 100,000 live births in 2017 globally, most sub-Saharan African (sSA) countries had difficulties in achieving the Millennium Development Goals (MDGs 4 and 5) [1, 2]. Similarly, the neonatal mortality rate declined by more than half (from 36.6 to 18 deaths per 1000 live births) between 1990 and 2017 globally [3]. MMRs and neonatal mortality rates, however, vary across regions. In 2018, 1 out of 37 neonates in sSA died compared to 1 in 333 in high income countries and MMR varied from 11 to 14 for high income regions to 511 to 652 per 100,000 live births for sSA [2–4]. Building on the MDGs, the United Nations member states adopted the Sustainable Development Goals (SDGs) to reduce the global MMR to less than 70 per 100,000 live births by 2030, with no country exceeding twice the global MMR (140 per 100,000 live births) [5]. Globally, 2.9 million newborns and 265,000 mothers die annually due to complications from pregnancy, childbirth and postpartum period. Of these, more than half occur in sSA [6]. Moreover, annually, an estimated 2.6 million stillbirths occur globally and 98% of all these stillbirths occur in low and middle income countries with three-fourths occurring in South Asia and sSA [7, 8].

Many strategies have been implemented in an attempt to reduce maternal and child deaths [9]. Continuum of Care (CoC) is one of the strategies that gained recent recognition and is advocated by the World Health Organization (WHO) and other initiatives to reduce stillbirth, neonatal, child and maternal mortality [6, 10]. Many maternal and neonatal deaths are largely preventable in low income countries by expanding the CoC approach [11–13].

Currently, CoC for maternal and newborn health (MNH) care is recommended as advantageous over each service provided separately to achieve the global target of ending preventable maternal, newborn and child deaths because each stage of the CoC builds on the success of the previous stages [6, 14, 15]. Lack of appropriate care at any stage of CoC leads to poor MNH outcomes [16].

Even though complete exposure to CoC has advantages over separate stages of care [17–20], evidence from the globe suggests that completion of CoC for maternal health is very low [6, 15, 21–31]. Many pregnant women (84.3%) received at least one ANC visit in sSA and South Asia, which varied from 43% in Ethiopia to 99% in Malawi. Only few women, however, utilize at least four ANC visits (37.9%) as previously recommended by WHO, had skilled birth attendance (28.3%) and received postnatal care (16.9%), indicating disparities in the coverage of each stage of maternal health care [32].

Several studies investigated associated factors of maternal health care utilization in sSA [33–36]. Most were mainly country-specific, with a primary focus on Ethiopia [23, 27, 28, 37, 38], Ghana [6, 21] and Nigeria [25, 29]. Moreover, very few studies have used nationally representative data [25, 36, 37]. Their narrow geographic scope limits generalizability, particularly when countries have complex multi-ethnic settings. To the best of our knowledge, little evidence exists on the status of CoC and its associated factors on a sSA scale. Therefore, it is essential to generate updated information on factors associated with CoC in order to design effective strategies to increase CoC. Instead of looking into the different stages of maternal health care, this study investigated CoC and factors that influence women’s continuation in receiving care because CoC is the most underutilized care and critical intervention to improve maternal and newborn wellbeing [28, 39].

## Methods

### Data source

All 33 Demographic and Health Surveys (DHSs), conducted in sSA from 2010 to 2018, were used. Only one DHS from Mozambique was excluded because it had no observations regarding PNC. We appended these datasets together to investigate completion and factors of CoC among women in sSA. DHSs are nationally representative surveys, conducted at five years’ intervals across low- and middle-income countries [40, 41]. Each country’s DHS follows a common execution procedure and uses the same definition of terms. It collects data on basic health indicators like sociodemographic characteristics, mortality, morbidity, family planning utilization, fertility and maternal and child health-related indicators.

A two-stage stratified cluster sampling procedure was used to select study participants. In the first stage, Enumerations Areas (EAs) were selected based on the sampling frame of each country. In the second stage, a sample of households is selected from each EAs. The detailed sampling procedure used by DHS has been documented elsewhere [42]. DHS surveys consist of five datasets; for this study we used the individual records’ data sets (IR file). IR files collected information from all eligible women aged 15–49 years. This study, however, was limited to women aged 15–49 years who had given birth within the last two years preceding the survey**.** This study used pooled data of the DHS surveys from 32 sSA countries. A total weighted sample of 225,135 women of reproductive age who gave birth was included.

### Study variables

#### Dependent variable

Completion of a CoC which was a composite score of ANC, SBA and PNC, was the dependent variable. It was dichotomized as complete if women had received at least four antenatal care visits (ANC4 +), SBA and PNC and incomplete if the women did not receive at least ANC4 + , SBA or PNC. Current (2016) WHO recommendations on ANC modified the minimum number of ANC contacts from four to eight. In this study, however, the frequency of ANC contacts was measured dichotomously as less than four ANC visits and at least four ANC visits since many DHS surveys were conducted before 2016 [43]. SBA was defined as births with the assistance of doctors, nurses, nurse/midwives, auxiliary midwives and others (health officers and health extension workers). PNC was defined as a health check-up by a health professional within 6 weeks of birth, coded as “1″ if women got postnatal care within 42 days and”0″ if otherwise [44, 45].

#### Independent variables

**Fig. 1:**
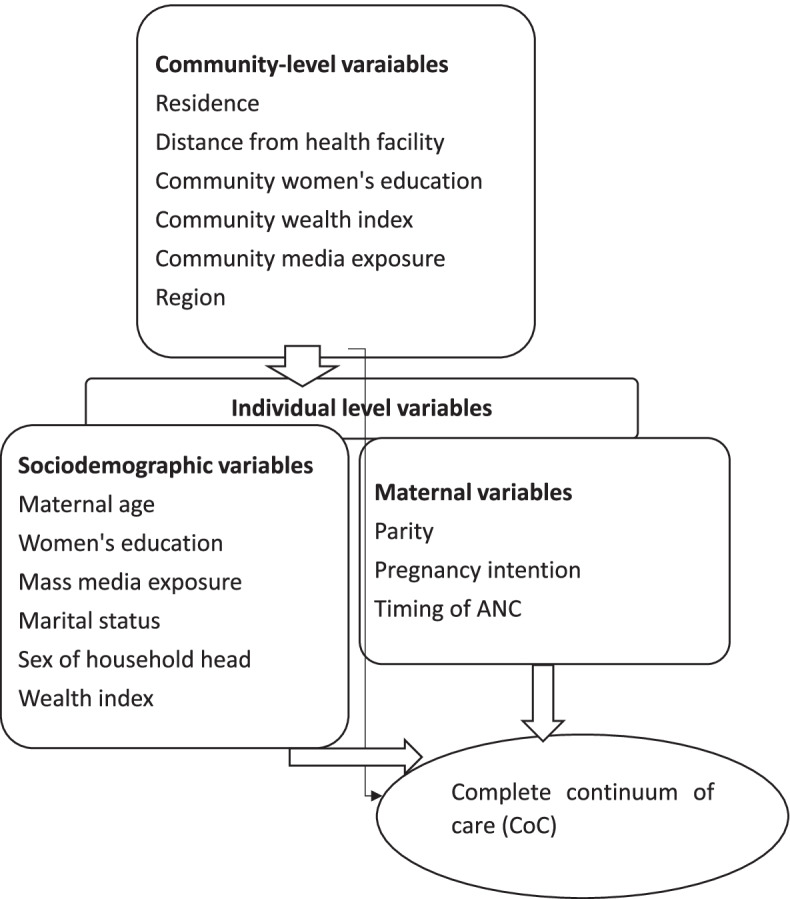
Conceptual framework on associated factors of* c*ompletion of continuum of care for maternal health among women

Based on previous literature [6, 15, 21–31, 46] and known facts, we considered both individual and community-level independent variables in assessing factors associated with CoC. These were sociodemographic factors (maternal age, women’s education, mass media exposure, marital status, sex of household head and household wealth quintiles) and maternal factors (parity, pregnancy intention and timing of ANC). Community-level variables were place of residence, distance from health facility, community women’s education, community wealth, community media exposure and region (Fig. 1). A detailed description and coding of variables have been documented in a DHS report (supplementary file 1).

### Data management and statistical analysis

Extraction, recoding and analysis of data were performed using Stata version 14 software. We summarized data by text, tables and figures. Data were weighted to assure representativeness of the survey and to get reliable statistical estimates (robust standard error). Weighting was done using the complex sample design weighting (sampling weight, primary sampling unit and strata) or “svyset” Stata command. We conducted a multilevel analysis since DHS data have been collected in nested units. For the purposes of the analysis, we fitted four models. First a null model without any explanatory variable was fitted to assess outcome variability between clusters. Then, model 1 was fitted with individual-level variables only and model 2 with community-level variables only. Finally, model 3 was fitted by incorporating both individual-level and community-level variables. Intra-class Correlation Coefficients (ICC), Median Odds Ratios (MOR), and the Proportional Change in Variance (PCV) were estimated to measure CoC variation across clusters. Model comparison was based on deviance (-2LLR) and the best-fit model was considered a model with the lowest deviance.

To select variables for multivariable analysis we fitted unadjusted regression models (bivariable analysis) for each independent variable. Variables with p-values ≤ 0.2 were selected for multivariable analysis (supplementary file 2). To declare the strength of association in the multivariable multilevel binary logistic regression, adjusted Odds Ratios (aOR) with 95% Confidence Interval (CI) of the best-fitted model were reported.

## Results

### Background characteristics of study participants

Out of a total of 225,135 women from 32 sSA countries, 161,948 (71.9%) had planned their recent pregnancy, 200,886 (89.2%) were currently in union and 122,301 (60.9%) initiated the first ANC visit after 12 weeks of gestation. Regarding educational status, 89,422 (39.7%) women had no education and 70,817 (31.5%) had completed primary level. Nearly two-thirds, 146,212 (65.0%) had exposure to at least one media during the week (radio, newspapers or television) and 150,447 (66.8%) were rural residents. The largest portion of the women who received antenatal care had their blood pressure measured (175,908; 87.5%) and received tetanus injection (172,982; 72.9%) (Table 1).

**Table 1:** Background Characteristics of study participants in sSA

Variables	Completion of CoC		Total (%)
	Yes (%)	No (%)	
Maternal age			
15–24	13,968 (20.7)	53,531 (79.3)	67,499 (30.0)
25–34	22,510 (21.8)	80,932 (78.2)	103,442 (45.9)
35–49	10,693 (19.7)	43,501 (81.3)	54,194 (24.1)
Women’s education			
Not educated	10,562 (11.8)	78,860 (88.2)	89,422 (39.7)
Primary	13,934 (19.7)	56,883 (80.3)	70,817 (31.5)
Secondary	19,029 (33.5)	37,752 (66.5)	56,781 (25.2)
Higher	3,646 (44.9)	4,469 (55.1)	8,115 (3.6)
Wealth status			
Poorest	6,168 (12.8)	41,897 (87.2)	48,065 (21.3)
Poorer	7,621 (16.0)	40,086 (84.0)	47,707 (21.2)
Middle	8,867 (19.5)	36,538 (80.5)	45,405 (20.2)
Richer	10,887 (24.7)	33,214 (75.3)	44,101 (19.6)
Richest	13,628 (34.2)	26,229 (65.8)	39,857 (17.7)
Marital status			
Currently in union	40,452 (20.1)	160,434, (79.9)	200,886 (89.2)
Not currently in union	6,719 (27.7)	17,530 (72.3)	24,249 (10.8)
Working status			
Not working	17,121 (20.7)	65,497 (79.3)	82,618 (32.7)
Working	30,018 (21.1)	112,278 (79.9)	142,296 (63.3)
Parity			
Primiparous	12,988 (27.8)	33,674 (72.2)	46,662 (20.7)
Multiparous	27,873 (21.6)	101,173 (78.4)	129,046 (57.3)
Grand multiparous	6,310 (12.8)	43,117 (87.2)	49,427 (22.0)
Pregnancy intention			
Intended	33,044 (20.4)	128,904 (79.6)	161,948 (71.9)
Unintended	14,120 (22.4)	49,019 (77.6)	63,138 (28.1)
Media exposure			
No	8,850 (11.3)	69,703 (88.7)	78,553 (35.0)
Yes	38,268 (26.2)	107,944 (73.8)	146,212 (65.0)
Timing of ANC			
Timely	26,197 (33.3)	52,481 (66.7)	78,678 (39.1)
Delayed	20,974 (17.1)	101,336 (82.9)	122,310 (60.9)
Sex of household head			
Male	34,995 (19.7)	142,701 (80.3)	177,696 (78.9)
Female	12,176 (25.7)	35,263 (74.3)	47,439 (21.1)
Blood pressure measured			
No	1,831 (7.3)	23,237 (92.7)	25,068 (12.5)
Yes	45,340 (25.8)	130,568 (74.2)	175,908 (87.5)
Blood sample has taken			
No	3,103 (9.7)	28,783 (90.3)	31,886 (15.9)
Yes	44,068 (26.1)	125,009 (73.9)	169,077 (84.1)
Urine sample was taken			
No	7,606 (12.8)	51,847 (87.2)	59,453 (29.6)
Yes	39,564 (28.0)	101,940 (72.0)	141,504 (70.4)
Received tetanus injection			
No	5,278 (10.1)	46,836 (89.9)	52,114 (23.1)
Yes	41,887 (24.2)	131,095 (75.8)	172,982 (72.9)
Iron supplementation			
No	3,105 (6.3)	46,183 (93.7)	49,288 (21.9)
Yes	44,065 (25.0)	131,775 (75.0)	175,840 (78.1)
Residence			
Urban	23,751 (31.8)	50,937 (68.2)	74,688 (33.2)
Rural	23,420 (15.6)	127,027 (84.5)	150,447 (66.8)
Distance from health facility			
Big problem	14,235 (16.3)	72,980 (83.7)	87,215 (40.3)
Not big problem	31,635 (24.5)	97,476 (75.5)	129,111 (59.7)

### Continuum of care for maternal health Care

**Fig. 2:**
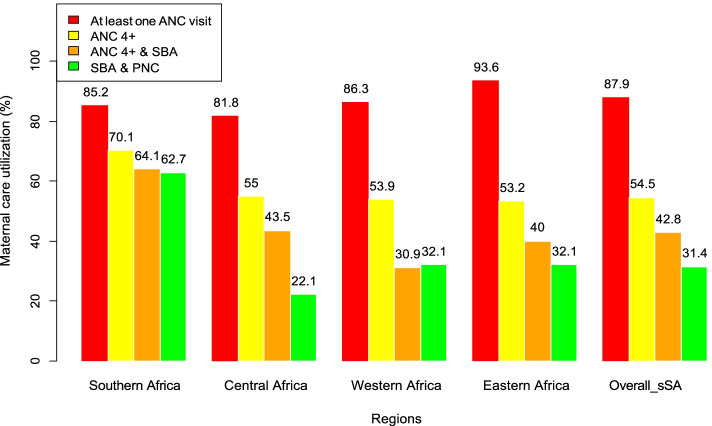
Utilization of different maternal health care among women by region

**Fig. 3:**
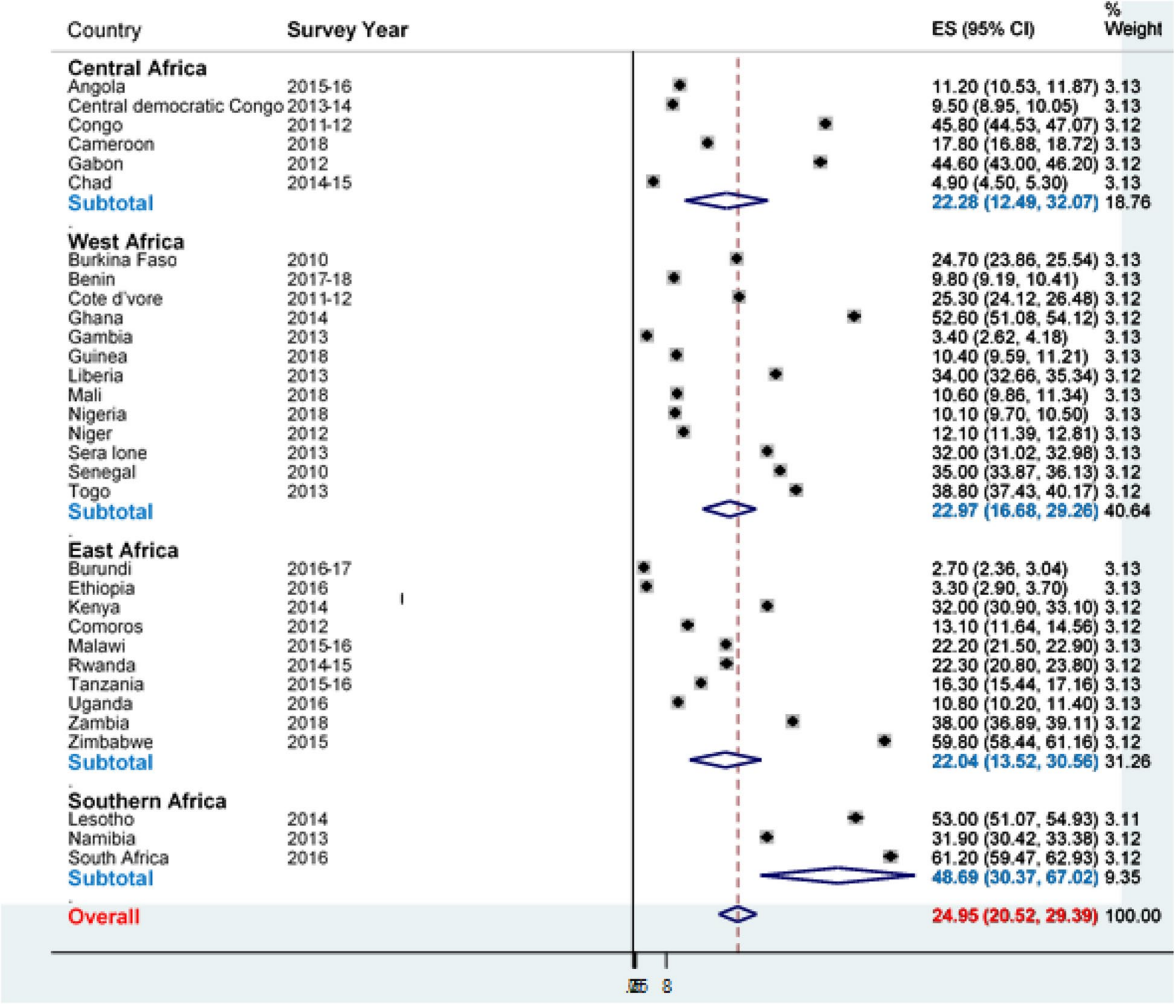
Magnitude of completion of maternal health care in 32 sub-Saharan African countries (2010–2018)

In sSA, 197,894 (87.9%) women attended at least one ANC visit during pregnancy. It was the commonest care received by women in each country which varied from 62.6% in Chad to 99.2% in Rwanda. The proportion of women with ≥ 4 ANC visits decreased to 54.5%, ranging from 31.1% in Chad to 87.2% in Ghana. The proportion of women who received ≥ 4 ANC visits and SBA was 42.8% (95% CI, 42.5, 43.1). The proportion of women who received SBA and PNC decreased to 31.4% (95 CI, 31.2, 31.6) (Fig. 2) and it ranged from 4.4% in Gambia to 78.9% in South Africa (Table 2). Only 56,284 (25.0%; 95% CI 20.5, 29.4) women received completed CoC (ANC4 + , SBA and PNC). It varied between the lowest of 17.9% (95% CI 10.1, 26.6) in East Africa to the highest of 51.5% (95% CI 37.5, 65.5) in Southern Africa (Fig. 3).

**Table 2:** Utilization of different maternal health care among women in 32 sSA countries

Country	At least one ANC visit	Number of ANC4 +	ANC4 + and SBA	SBA and PNC
	Frequency (%)	Frequency (%)	Frequency (%)	Frequency (%)
**Southern Region of Africa**				
Lesotho	2,439 (94.7)	1,917 (74.4)	1,687 (65.5)	1,609 (62.5)
Namibia	2,828 (74.1)	2,402 (62.9)	2,203 (57.8)	1,908 (49.8)
South Africa	2,762 (93.7)	2,292 (75.0)	2,231 (73.5)	2,395 (78.9)
Zimbabwe	4,652 (93.3)	3,777 (75.7)	3,323 (66.6)	3,637 (73.0)
**Central Region of Africa**				
Angola	6,875 (80.9)	5,219 (61.4)	3,358 (39.5)	1,127 (13.3)
DR Congo	9,885 (89.4)	5,310 (48.0)	4,711 (42.7)	1,798 (16.3)
Congo	5,448 (92.6)	4,641 (78.9)	4,459 (75.8)	3,194 (54.3)
Cameroon	5,698 (86.2)	4,289 (64.9)	3,617 (54.7)	1,413 (21.4)
Gabon	3,462 (93.5)	2,873 (77.6)	2,778 (75.0)	1,994 (53.9)
Chad	6,958 (62.6)	3,452 (31.1)	1,443 (13.0)	836 (7.5)
**Eastern Region of Africa**				
Burundi	8,873 (99.2)	4,404 (49.3)	3,855 (43.1)	423 (4.8)
Ethiopia	4,756 (62.7)	2,415 (31.8)	1,359 (17.9)	362 (4.8)
Kenya	13,824 (95.7)	8,319 (57.6)	6,235 (43.3)	3,262 (47.5)
Malawi	13,218 (97.8)	6,836 (50.6)	6,554 (48.5)	5,563 (41.3)
Rwanda	6,010 (99.2)	2,663 (44.0)	2,543 (42.0)	1,416 (47.8)
Tanzania	6,902 (97.5)	3,588 (50.7)	2,738 (38.7)	1,956 (22.6)
Comoros	1,615 (78.2)	1,009 (48.9)	842 (40.8)	551 (26.7)
Uganda	9,901 (97.5)	6,080 (59.9)	5,006 (49.3)	1,581 (15.6)
Zambia	7,182 (98.0)	4,651 (63.5)	4,198 (57.3)	4,132 (56.4)
**Western Region of Africa**				
Burkina-Faso	9,966 (95.0)	3,531 (33.7)	3,034 (28.9)	6,282 (62.1)
Benin	7,814 (86.5)	4,701 (52.1)	4,498 (49.8)	1,483 (16.4)
Cote d’Ivoire	4,798 (91.6)	2,316 (44.2)	1,826 (35.0)	2,285 (43.8)
Ghana	4,014 (96.9)	3,614 (87.2)	2,907 (70.2)	2,293 (55.4)
Gambia	5,252 (99.0)	4,119 (77.6)	2,778 (52.5)	90 (4.4)
Guinea	4,572 (83.3)	1,936 (35.3)	1,384 (25.2)	1,137 (20.8)
Liberia	4,492 (95.2)	3,726 (78.1)	2,469 (51.8)	1,890 (39.6)
Mali	5,188 (78.3)	2,864 (43.3)	2,489 (37.6)	1,159 (17.6)
Nigeria	16,217 (67.0)	12,456 (56.8)	7,673 (35.0)	2,557 (11.7)
Niger	6,817 (85.2)	2,623 (32.8)	1,210 (15.1)	2,113 (26.4)
Sierra Leone	7,368 (85.2)	6,573 (76.0)	3,924 (45.4)	3,416 (39.5)
Senegal	7,190 (93.6)	3,840 (50.0)	3,311 (43.0)	4,037 (58.8)
Togo	4,487 (92.4)	2,777 (57.2)	2,431 (50.1)	2,790 (57.5)

### Multilevel analysis

#### Random parameter estimation and model selection

The null model in the multilevel analysis indicates 6.8% of the total variation on maternal health care along the CoC was at cluster level and may be attributable to community-level factors. Additionally, the null model had the highest MOR value (1.59) indicating the effects of community heterogeneity were high as compared with the final model (model 3). In model 3, as indicated by the PCV, 50% of the variation in maternal health care along the CoC across communities was explained by both individual and community-level factors. Also, model fitness was compared using deviance and the model with the lowest deviance (model 3) was considered the best-fitting model (Table 3).

**Table 3:** Individual and community-level factors associated with continuum of care for maternal health in Sub-Saharan Africa

Variables	Null model	Mode 1 aOR (95% CI)	Model 2 aOR (95% CI)	Model 3 aOR (95% CI)
Maternal age				
15–24	-	1	-	1
25–34	-	1.27(1.22,1.29)	-	1.22 (1.17,1.25)
35–49	-	1.56(1,49,1.61)	-	1.40(1.35,1.47)
Women’s education	-			
Not educated	-	1	-	1
Primary	-	1.49(1.43,1.54)	-	1.44 (1.41,1.49)
Secondary	-	2.12(2.04,2.17)	-	1.95 (1.89,2.03)
Higher	-	2.38(2.24,2.53)	-	2.15 (2.01,2.25)
Wealth status				
Poorest	-		-	1
Poorer	-	1.02(0.98,1.03)	-	0.98 (0.96,1.02)
Middle	-	1.04(0.98,1.08)	-	1.01 (0.98,1.05)
Richer	-	1.09(1.07,1.13)	-	1.02 (0.97,1.06)
Richest	-	1.12(1.09,1.16)	-	1.04 (0.99,1.08)
Marital status				
Currently in union	-	1	-	1
Not currently in union	-	1.01(0.98,1.04)	-	0.98 (0.96,1.04)
Working status				
Not working	-	1	-	1
Working	-	0.98(0.96,1.03)	-	1.01 (0.98,1.04)
Parity				
Primiparous	-	1	-	1
Multiparous	-	0.89(0.87,0.92)	-	0.97 (0.95,1.03)
Grand multiparous	-	0.75(0.73,0.78)	-	0.99 (0.95,1.04)
Pregnancy intention				
Intended	-	1	-	1
Unintended	-	1.11(1.08,1.14)	-	0.87 (0.84,0.91)
Media exposure				
No	-		-	1
Yes	-	1.39(1.37,1.44)	-	1.35 (1.28,1.39)
Timing of ANC				
Timely	-	1	-	1
Delayed	-	0.51(0.48,0.54)	-	0.43 (0.41,0.47)
Sex of household head				
Male	-	1	-	1
Female		1.13(1.11,1.16)	-	1.18 (1.15,1.21)
Residence				
Urban	-	-	1	1
Rural	-	-	0.50(0.48,0.50)	0.78 (0.75,0.81)
Distance from health facility				
Not big problem	-	-	1	1
Big problem	-	-	0.72(0.71,0.74)	0.88 (0.85,0.91)
Community education				
Low	-	-	1	1
High	-	-	0.99(0.92,1.06)	1.12 (1.09,1.16)
Community wealth				
Low	-	-	1	1
High	-	-	0.95 (0.89,1.02)	1.02 (0.97,1.07)
Community media exposure				
Low	-	-	1	1
High	-	-	0.79(0.74,0.85)	0.97 (0.94,1.05)
Region				
Western Africa			1	1
Southern Africa			3.01(2.87,3.16)	2.03 (1.99,2.11)
Central Africa			0.89 (0.87,0.92)	0.94 (0.91,1.02)
Eastern Africa			1.04 (0.98,1.07)	1.01 (0.98,1.05)
Random parameters and model comparison				
Community level variance	0.24	0.11	0.22	0.08
ICC (%)	6.80	3.23	6.27	2.27
MOR	1.59	1.37	1.56	1.31
PCV (%)	Ref	54.2	8.3	66.7
DIC (-2LLR)	227,149.7	189,798.8	211,527.0	182,942.3

#### Factors associated with the CoC completion in sSA

The odds of completing CoC were 1.22 (aOR 1.22; 95% CI 1.17, 1.25) among women aged 25–34 years and 1.40 (aOR 1.40; 95% CI 1.35, 1.47) among ≥ 35 years as compared to women aged 15–24 years. The odds of completing CoC were 1.44 (aOR 1.44; 95% CI 1.41, 1.49) for mothers who had primary education, 1.95 (aOR 1.95; 95% CI 1.89, 2.03) for secondary education and 2.15 (aOR 2.15; 95% CI 2.01, 2.25) for higher education as compared to those without formal education. Moreover, the odds of completing CoC was higher among women who headed female households (aOR 1.18; 95% CI 1.15, 1.21), were exposed to mass media (aOR 1.35; 95% CI 1.28,1.39) and with high community education (aOR 1.12; 95% CI 1.09,1.16).

The odds of completing CoC among women who reside in rural areas were 22% lower (aOR 0.78; 95% CI 0.75, 0.81) as compared with women who reside in urban areas. The odds of completing CoC among women reporting distance to a health facility as the big problem was decreased by 12% (aOR 0.88; 95% CI 0.85, 0.91) compared with women who deem distance to a health facility not a big problem. Similarly, women who did not intended to be pregnant had lower odds of completing CoC as compared to those with planned pregnancies (aOR 0.87; 95% CI 0.84, 0.91). In addition, women who started ANC after 12 weeks of gestation had lower odds of completing CoC as compared to those who initiated ANC before 12 weeks of gestation (aOR 0.43; 95% CI 0.41, 0.47) (Table 3).

## Discussion

Completion of CoC occurred in only 56,284 (25.0%, 95% CI 20.5, 29.4) of the women in sSA. This was higher in Southern Africa with 51.5% (95% CI 37.5, 65.5) and much lower with approximately 20% in the other three regions. Large disparities of CoC were found across sSA countries with as low as 2.7% in Burundi and as high as 61.2% in South Africa. This finding is in line with pooled estimates of South Asian countries (24.5%) [32]. Our finding, however, is higher than the pooled estimates of a previous study in sSA (13.9%) [32]. The higher completion rate in this study might be attributed to the inclusion of recent and most DHS in sSA, while that study included only five East African countries and one West African country [32]. Possibly maternal health care utilization has improved substantially over time in several sSA countries [47]. Further, this discrepancy might be due to differences in timing of ANC initiation, since timing of ANC is one of the predictors for completion of the CoC [23, 48] and in our study, 39.1% of women initiated ANC within the first 12 weeks of gestation, higher than in the previous one (23.9%). Even though focused ANC includes at least four ANC visits, SBA and at least three postnatal care visits for all mothers and newborns, this comprehensive analysis of 32 DHS from sSA showed low proportions of women utilizing CoC [49, 50].

This study demonstrated that the inclusion of community-level variables was important in explaining the variations in the completion of CoC. Community-level variables such as region, residence, distance from health facilities and community education showed effects on the completion of maternal health care. Only 2.27% of the total variation remained unexplained after adjustments of individual and community level factors.

When maternal age increases, the odds of completing CoC increases, consistent with some previous studies [51, 52]. Some others, however, showed different effects [6, 22–24, 27]. One study indicated that younger women were less likely to recognize pregnancy early [53]. It is also observed in that study, that 22.5% of women < 25 years were never married compared with 5.0% of women aged 25–34 years and 2.2% ≥ 35 years and most unintended pregnancies were among unmarried women. Younger women who are unmarried are not likely to disclose pregnancy to avoid potential social implications since premarital pregnancies are highly stigmatized [54]. Risks of maternal and fetal complications are, however, more likely among adolescent women and lower utilization of maternal health care by adolescents is particularly problematic [55, 56].

Consistent with studies in Ethiopia, Nigeria, Ghana, Chad, Gambia, Nepal, Pakistan, higher women’s education was significantly associated with increased odds of CoC [22–25, 28, 30, 32, 36, 51, 52]. Women’s understanding of multiple dimensions of health and health knowledge increased with more education, leading women to seek greater access of acceptable maternal and new born care [57]. Moreover, educated women are more aware of health-protective information and have greater decision-making power and demand higher quality care and pay more attention to their health in order to ensure better health for themselves and their children [58].

Supported by studies conducted in Ethiopia, Chad, Pakistan and Nepal, women exposed to mass media were more likely to utilize CoC [22, 24, 27, 36, 46, 59, 60]. Mass media are important means of disseminating information concerning maternal health, increasing knowledge, attitude and behavior of women towards maternal health care utilization [61–63].

As observed in previous studies, lower odds of completing CoC were observed among women in rural areas as compared to urban areas [24, 25, 28, 29, 31, 36, 37, 46, 59, 66]. Lack of adequate health facilities, a deficit in health care professionals, lack of infrastructure (e.g. road conditions, costs of transport), distance from health facilities are more prevalent in rural areas [22, 64]. Moreover, women residing in urban areas have better educational status and better chances of health information than rural women. Therefore, provision of maternal health care to rural women through home visits, outreach programs and mass media campaigns should be implemented to improve CoC [65].

Women with unintended pregnancies had lower odds of completing CoC as compared to planned pregnancies, similar to studies in Ethiopia, Ghana and Nepal [6, 25, 29, 30]. Women with intended pregnancies are more likely to detect pregnancy earlier and may be careful about their pregnancy status compared to those with unintended pregnancies [66]. Moreover, women with unintended pregnancies are less likely prepared emotionally and financially for the demands of pregnancy and childbearing and less likely to care for the baby [67].

Delayed initiation of ANC was negatively and significantly associated with utilization of CoC, in agreement with studies from Ethiopia, Gambia, and Japan [15, 23, 36, 48]. Early initiation of ANC gives the opportunity to discuss birth preparedness and complication readiness and helps women to receive health promotion and preventive care such as immunization against tetanus, nutrition counseling, prophylactic treatment of malaria and worms [68–70]. Therefore, this study indicated that timely initiation of ANC and quality of ANC is vital to completion of maternal health care along the CoC.

The odds of completing CoC was found to be higher among women from communities with a high percentage of educated women. Low literacy levels in the community may be related to low health knowledge, while women from highly educated communities may acquire information from others regarding benefits of maternal health care, leading to increased odds of completing CoC. Illiterate women are economically unstable and may fail to receive adequate maternal health care during pregnancy [71].

### Strengths and limitations

Strengths of this study are first of all large sample size from nationally representative data from 32 sSA countries with appropriate multilevel statistical analysis. Second, our study applied a more comprehensive measure of maternal health care that collectively considered access to CoC. Our findings, however, cannot provide information on causality as our study had a cross-sectional design. Measurement of the main components of CoC is self-reported based on women’s recall and this may have led to recall and social desirability bias. Due to the secondary nature of data, we did not include some factors which may influence utilization along the CoC, such as quality and satisfaction with care, knowledge of maternal health care and danger signs. Besides, it was unable to assess complete PNC within six weeks after birth of women and newborns as an element of CoC because such data were not collected in DHSs.

## Conclusion

Low proportions of women utilizing CoC in sSA were observed with the highest coverage in Southern Africa and almost similar low coverage in East, West and Central Africa. Both individual and community-level factors were associated with CoC completion. Factors associated with high CoC completion rates, include older age, having attended education, having mass media exposure, intended pregnancy, timely ANC initiation, female-headed households,, perceiving distance from health facilities not as big problem and residing in urban areas and from highly educated communities. These findings point to areas where care can be better tailored to improve the completion of CoC. Policymakers in sSA must consider both individual and community-level factors and undertake multi-sectorial approaches to address barriers at different levels. Thus, those living in rural areas, less educated, initiated ANC lately, perceiving distance from health facilities as big problem and from communities with low education need more attention to increase completion of CoC and improve maternal and newborn health.

## Supplementary Information

**Additional file 1.**

**Additional file 2.**

## Data Availability

Datasets for this study were existing public domain survey data which were accessed from http://www.dhsprogram.com.

## Abbreviations

ANC: Antenatal Care
aOR: Adjusted Odds Ratio
CI: Confidence Interval
CoC: Continuum of Care
DHS: Demographic and Health Survey
ICC: Intra-class Correlation Coefficient
MOR: Median Odds Ratio
PCV: Proportional Change in Variance
PNC: Postnatal Care
SBA: Skilled birth Attendance
sSA: Sub-Saharan Africa
