# Supplementary material for: Coverage and factors associated with completion of continuum of care for maternal health in sub-Saharan Africa: a multicountry analysis
Source: BMC Pregnancy Childbirth. 2022 May 19;22:422. doi: 10.1186/s12884-022-04757-1 (PMC9121540; doi:10.1186/s12884-022-04757-1)
Supplement: Supplementary file 1 — Additional file 1. [file 12884_2022_4757_MOESM1_ESM.docx]

**Supplementary file 1**: measurement of outcome and independent variables used in the analysis among women with most recent births for 32 sub-Saharan African countries

| Variables | Description/definitions (coding categories) |
| --- | --- |
| PNC | A health check-up by a health professional within 6 weeks of birth, coded as “1” if women got postnatal care within in 42 days and”0″ otherwise. |
| SBA | Births with the assistance of doctors, nurses, nurse/midwives, auxiliary midwives, and others (health officers and health extension workers), coded as “1” if women gave birth with the assistance of health professionals and”0″ otherwise. |
| CoC | A composite score of ANC, SBA and PNC. It was dichotomized as complete if women had received at least four antenatal care visits (ANC4+), SBA and PNC and incomplete if the women did not receive at least ANC4+, SBA or PNC. |
| maternal age | Mother’s age at interview (15-24 years, 25-34 years, or 35-49 years) |
| Women’s education | Highest education level attained by respondents (no education, primary , secondary or higher) |
| Mass media exposure | Composite of listening radio, reading magazine/newspaper, and watching television. It is coded as exposed if women are exposed to at least one medium (radio, magazine/newspaper, or television) at least once a week and ‘non-exposed’ if they are not exposed |
| marital status | Currently in union/not currently in union) |
| sex of household head | Female/male |
| wealth index | Measure of household wealth status based on household assets (poorest, poorer, poor, richer or richest) |
| Parity | Total number of children ever born and abortion (recoded as primiparous (1 birth), multiparous (2-5 births), or grand multiparous (>5 births) |
| pregnancy intention | Wanted pregnancy when became pregnant for most recent birth (intended or unintended) |
| Working status | Working/not working) |
| Timing of ANC | Dichotomized as: if first ANC visit was within 12 weeks of gestation as “timely”, or after 12 weeks of gestation as ”delayed” |
| Residence | Urban/rural |
| Distance from health facility | Self-report of respondents on perception of distance from the health facility (big problem/not big problem) |
| Community education | Measured as the proportion of women with at least primary education in the primary sampling unit. Categorized as low and high, based on median. |
| community media exposure | The proportion of women exposed to at least one type of media; radio, newspaper or television in the primary sampling unit. Categorized as low and high, based on median. |
| Community wealth | Computed from the household wealth and defined as   the proportion of women in the poorest and poorer quintiles in the community. Categorized as low and high based on median. |
| Region | Based on UN sub-region classification (Southern Africa, Western Africa, Central Africa or Eastern Africa) |

Note: CoC; Continuum of Care, PNC; Postnatal Care, SBA; Skilled Birth Attendant
