# Supplementary material for: Coverage and factors associated with completion of continuum of care for maternal health in sub-Saharan Africa: a multicountry analysis
Source: BMC Pregnancy Childbirth. 2022 May 19;22:422. doi: 10.1186/s12884-022-04757-1 (PMC9121540; doi:10.1186/s12884-022-04757-1)
Supplement: Supplementary file 2 — Additional file 2. [file 12884_2022_4757_MOESM2_ESM.docx]

Supplementary file 2: Bivariable analysis of factors associated with continuum of care for maternal health in Sub-Saharan Africa

| Variables | Crude odds ratio (95% CI) | Variables | Crude odds ratio (95% CI) |
| --- | --- | --- | --- |
| Maternal age |  | Timing of ANC |  |
| 15-24 | 1 | Timely | 1 |
| 25-34 | 1.04 (1.02,1.07) | Delayed | 0.43 (0.42,0.44) |
| 35-49 | 0.93(0.90,0.95) | sex of household head |  |
| Women’s education |  | Male | 1 |
| Not educated | 1 | Female | 1.37 (1.33,1.40) |
| Primary | 1.85 (1.80,1.90) | Residence |  |
| Secondary | 3.62 (3.52,3.72) | Urban | 1 |
| Higher | 5.82 (5.52,6.13) | Rural | 0.45 (0.44,0.46) |
| Wealth status |  | Distance from health facility |  |
| Poorest | 1 | Not big problem | 1 |
| Poorer | 1.25 (1.21,1.30) | Big problem | 0.60 (0.58,0.61) |
| Middle | 1.52 (1.47,1.58) | Community education |  |
| Richer | 2.01 (1.94,2.08) | Low | 1 |
| Richest | 2.97 (2.87,3.07) | High | 0.80 (0.75,0.85) |
| Marital status |  | Community wealth |  |
| Currently in union | 1 | Low | 1 |
| Not currently in union | 0.86 (0.84,0.92) | High | 0.98 (0.93,1.02) |
| Working status |  | Community media exposure |  |
| Not working | 1 | Low | 1 |
| Working | 1.04 (1.02,1.06) | High | 0.91(0.87,0.95) |
| Parity |  | Region |  |
| Primiparous | 1 | Western Africa | 1 |
| Multiparous | 0.93 (0.91,0.99) | Southern Africa | 3.70 (3.54,3.86) |
| Grand multiparous | 0.88 (0.83,0.94) | Central Africa | 0.72 (0.70,0.74) |
| Pregnancy intention |  | Eastern Africa | 1.07 (1.04,1.10) |
| Wanted | 1 |  |  |
| Unwanted | 1.04 (1.01,1.07) |  |  |
| Media exposure |  |  |  |
| No | 1 |  |  |
| Yes | 1.58 (1.52,1.65) |  |  |
